# Air pollution, physical activity and health: A mapping review of the evidence

**DOI:** 10.1016/j.envint.2020.105954

**Published:** 2021-02

**Authors:** Marko Tainio, Zorana Jovanovic Andersen, Mark J. Nieuwenhuijsen, Liang Hu, Audrey de Nazelle, Ruopeng An, Leandro M.T. Garcia, Shifalika Goenka, Belen Zapata-Diomedi, Fiona Bull, Thiago Herick de Sá

**Affiliations:** a Sustainable Urbanisation Programme, Finnish Environment Institute SYKE, Helsinki, Finland; b Systems Research Institute, Polish Academy of Sciences, Warsaw, Poland; c Department of Public Health, University of Copenhagen, Copenhagen, Denmark; d ISGlobal - Barcelona Institute for Global Health, Barcelona, Spain; e Universitat Pompeu Fabra, Barcelona, Spain; f CIBER Epidemiología y Salud Pública, Madrid, Spain; g Department of Sport Science, Zhejiang University, Hangzhou, China; h Centre for Environmental Policy, Imperial College London, London, UK; i Brown School, Washington University in St. Louis, St. Louis, US; j Centre for Public Health, Queen’s University Belfast, Belfast, UK; k Centre for Chronic Disease Control and Public Health Foundation of India, New Delhi, India; l Centre for Urban Research, RMIT University, Melbourne, Australia; m Department of Health Promotion, World Health Organization, Geneva, Switzerland; n Department of Environment, Climate Change and Health, World Health Organization, Geneva, Switzerland

**Keywords:** Air pollutants, Exercise, Active travel, Transport, Environment and public health

## Abstract

**Background:**

Exposure to air pollution and physical inactivity are both significant risk factors for non-communicable diseases (NCDs). These risk factors are also linked so that the change in exposure in one will impact risks and benefits of the other. These links are well captured in the active transport (walking, cycling) health impact models, in which the increases in active transport leading to increased inhaled dose of air pollution. However, these links are more complex and go beyond the active transport research field. Hence, in this study, we aimed to summarize the empirical evidence on the links between air pollution and physical activity, and their combined effect on individual and population health.

**Objectives and methods:**

We conducted a non-systematic mapping review of empirical and modelling evidence of the possible links between exposure to air pollution and physical activity published until Autumn 2019. We reviewed empirical evidence for the (i) impact of exposure to air pollution on physical activity behaviour, (ii) exposure to air pollution while engaged in physical activity and (iii) the short-term and (iv) long-term health effects of air pollution exposure on people engaged in physical activity. In addition, we reviewed (v) public health modelling studies that have quantified the combined effect of air pollution and physical activity. These broad research areas were identified through expert discussions, including two public events performed in health-related conferences.

**Results and discussion:**

The current literature suggests that air pollution may decrease physical activity levels during high air pollution episodes or may prevent people from engaging in physical activity overall in highly polluted environments. Several studies have estimated fine particulate matter (PM_2.5_) exposure in active transport environment in Europe and North-America, but the concentration in other regions, places for physical activity and for other air pollutants are poorly understood. Observational epidemiological studies provide some evidence for a possible interaction between air pollution and physical activity for acute health outcomes, while results for long-term effects are mixed with several studies suggesting small diminishing health gains from physical activity due to exposure to air pollution for long-term outcomes. Public health modelling studies have estimated that in most situations benefits of physical activity outweigh the risks of air pollution, at least in the active transport environment. However, overall evidence on all examined links is weak for low- and middle-income countries, for sensitive subpopulations (children, elderly, pregnant women, people with pre-existing conditions), and for indoor air pollution.

**Conclusions:**

Physical activity and air pollution are linked through multiple mechanisms, and these relations could have important implications for public health, especially in locations with high air pollution concentrations. Overall, this review calls for international collaboration between air pollution and physical activity research fields to strengthen the evidence base on the links between both and on how policy options could potentially reduce risks and maximise health benefits.

## Highlights

• Air pollution (AP) and physical activity (PA) are important health risk factors;
• We reviewed current evidence of AP and PA interactions for health;
• PA behaviour and health effects might be moderated by AP exposure;
• Epidemiological studies provide mixed results on AP and PA interaction;
• More research collaboration is needed to study AP and PA relations.

## 1 Introduction

Globally more than a quarter of adults are physically inactive, meaning that they do not achieve the minimum recommended level of 150 min of moderate-intensity or 75 min of vigorous-intensity physical activity per week, with adults from high-income countries being more inactive than adults from low- and middle-income countries (LMICs) (Guthold et al., 2018). At the same time, 90% of the global population lives in environments that exceed the World Health Organization (WHO) guideline values for ambient air quality, with LMICs countries suffering the greatest health burden (WHO, 2019a). Worldwide, WHO estimated that ambient air pollution caused 4.2 million deaths in 2016, while physical inactivity was estimated to cause 3.2 million deaths for the same year (WHO, 2019b).

Both air pollution and physical inactivity are causally and positively associated with premature mortality from non-communicable diseases (NCDs). Physical activity has immediate beneficial effects, which accumulate over time, and in the long run reduces the risk of developing and dying from cardiovascular and respiratory diseases, type 2 diabetes, certain types of cancers, and reduces the risk of all-cause mortality (Johnsen et al., 2013, Samitz et al., 2011, Schnohr et al., 2006, Woodcock et al., 2011). Exposure to air pollution increases the risk of cardiovascular and respiratory diseases, type-2-diabetes, and cancers, and premature mortality in the long term (Beelen et al., 2013, Hoek et al., 2013, Newby et al., 2015, Brook et al., 2017).

**Fig. 1:**
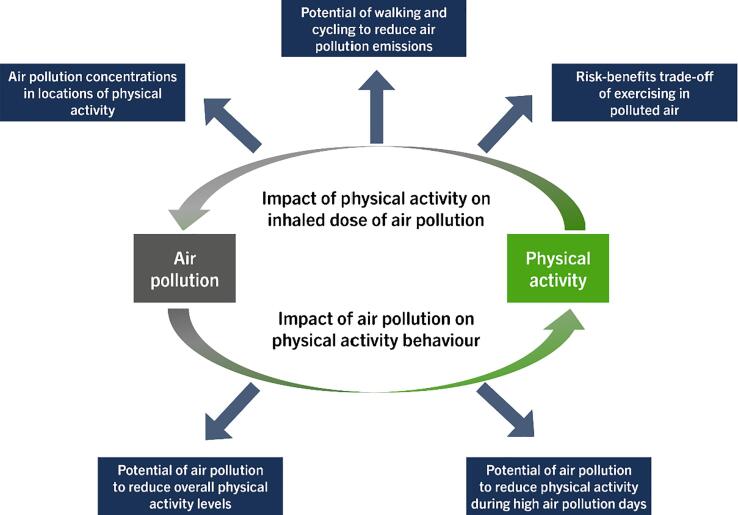
Schematic overview of the air pollution and physical activity interactions.

There are other links between air pollution and physical activity through antagonistic mechanisms that need consideration. For example, air pollution may impact physical activity behaviour and may negate some or all the benefits of doing physical activity (Fig. 1). These impacts could have important implications for public health, especially in highly polluted locations. For example, in China, researchers and policy makers have recognised that outdoor ambient air pollution could create a barrier for doing outdoor physical activity, and that the health risk from exposure to air pollution could negate the benefits of policies aimed at increasing physical activity (Wang, 2016 Sep, Si and Cardinal, 2017, Li et al., 2015 Mar). However, the impact of air pollution on physical activity behaviour and health (directly and indirectly by preventing people from doing physical activity) is not limited only to highly polluted outdoor environments; Outdoor air pollution also penetrates to indoor environments, and at the same time indoor environments have their own sources of air pollution (e.g. Martins and da Graca, 2018). Hence, air pollution in all environments (indoors and outdoors) may impact physical activity behaviour and health in multiple domains (transport, leisure, occupation and leisure).

This mapping review presents a summary of studies that identify the public health links between air pollution and physical activity. The main aim is to provide a synthesis of the inter-relationship between air pollution and physical activity and their combined effects on health. The specific objectives of this review are to summarise the research evidence on (a) the influence of air pollution on physical activity behaviour, (b) the exposure to air pollution while being physically active, (c) the health effects of short- and long-term exposure to air pollution while being physically active, and (d) the potential public health effects of the combined exposure. This report primarily focuses on ambient air pollution, especially fine particulate matter (PM_2.5_), ozone (O_3_) and nitrogen dioxide (NO_2_), as these pollutants have the largest public health relevance worldwide. We also focused on the transport and leisure domains of physical activity, considering that these domains are more easily modifiable.

## 2 Methods

This study is a non-systematic mapping review of the empirical and modelling evidence on the possible links between exposure to air pollution and physical activity. Our thinking on the review of these possible links between air pollution and physical activity was initially informed by reflection on a series of discussions and meetings among the authors. We further developed this thinking and refined the research areas with the discussions from two public events - one symposium in the 2018 Joint Annual Meeting of the International Society of Exposure Science and the International Society for Environmental Epidemiology (ISES-ISEE) in Ottawa, Canada, and one session on the 2018 congress of the International Society for Physical Activity and Health (ISPAH) in London, UK.

Five broad research areas on the links between air pollution and physical activity were identified (see Fig. 1) for the mapping review, which are as follows: (i) impact of exposure to air pollution on physical activity behaviour, (ii) exposure to air pollution while engaging in physical activity, (iii) short-term and (iv) long-term health effects of air pollution exposure while engaging in physical activity, and (v) public health modelling studies that have quantified the combined effect of air pollution and physical activity. Databases searched included MEDLINE, Scopus and Google Scholar. The final mapping review of the links between air pollution and physical activity was conducted in Autumn 2019.

The subsequent sections (sections 3 to 7) will present the results and further detail the evidence found for each of the above research areas identified.

## 3 Impact of air pollution on physical activity behaviour

The adverse effects of air pollution on disease outcomes have been extensively documented (WHO 2018), but less is known regarding its impact on people’s behaviour itself. Ambient air pollution may discourage people from engaging in regular physical activity through several mechanisms. First, air pollution decreases lung function, elevates blood pressure, and other cardiovascular and respiratory symptoms (Auchincloss et al., 2008, Brook et al., 2010, Cakmak et al., 2011) resulting in impaired exercise capacity and performance (Cutrufello et al., 2011, Marr and Ely, 2010, Rundell and Caviston, 2008). Second, visible air pollution, such as smog, could discourage people from engaging in outdoor activities (Roberts et al., 2014). In what follows, we summarise recent evidence of the impact of air pollution on physical activity behaviour for populations living in highly polluted environment and on physical activity behaviour during episodes of high air pollution. Key studies are summarized in Table 1.

**Table 1:** Summary of studies (from oldest to newest) that examined impact of air pollution on physical activity in An et al. (2018) and An et al. (2019) reviews.

**Study**	**Country**	**Study design**	**Sample size**	**Age (years)**	**Air pollution measure**	**Main findings**
Wen et al. (2009b)	U.S.	Cross-sectional	33,888	≥18	Air quality index	The prevalence of change in outdoor activity due to media alerts of AQI was 31% among adults with lifetime asthma and 16% without asthma. The prevalence of outdoor activity change increased to 75% among those with lifetime asthma and to 68% without asthma, when the combined the effects of media alerts and individual perception were examined. The odds of activity change based on the media alerts was 2.30 (OR = 2.16, 95% CI = 1.61, 2.90) among those with lifetime asthma and 1.72 (OR = 1.72, 95% CI = 1.50, 1.98) without asthma, compared to those unaware of media alerts, after adjustment for demographic variables and covariates.
Wen et al. (2009) Feb	U.S.	Cross-sectional	63,290	≥18	PM_2.5_	A 10-unit (μg/m^3^) increase in county annual average PM_2.5_ concentration was found to be associated with an increase in the odds of physical inactivity by 16% (OR = 1.16, 95% CI = 1.06, 1.27).
Hankey et al. 2012	U.S.	Cross-sectional	30,007	38 (range: 21–54)	PM_2.5_, O_3_, NO_x_	Between-neighborhood differences in the estimated risk for ischemic heart disease (IHD) mortality from air pollution were comparable in magnitude (9 more risk for IHD deaths/100,000/year for PM_2.5_ and 3 fewer IHD deaths for O_3_ in high- vs. low-walkability neighborhoods), suggesting that population health benefits from increased physical activity in high-walkability neighborhoods may be offset by adverse effects of air pollution exposure.
Wells et al. 2012	U.S.	Cross-sectional	10,898	46.9 (95% CI: 46.3, 47.6)	General air quality	A total of 1305 (12.0%, 95% CI = 10.9, 13.1) individuals responded that they did something differently due to bad air quality. Among those who reported changing their activity, the most commonly reported change was to spend less time outdoors.
Roberts et al. 2014	U.S.	Cross-sectional	329,628	≥18	PM_2.5_, PM_10_, O_3_	A 2.4% relative increase in the odds of physical inactivity per mg/m^3^ increase of PM_2.5_ exposure among the obese respondents (OR = 1.02, 95% CI = 1.00,1.05). An increasing concentration of PM_10_ among the normal weight respondents was also associated with higher odds of inactivity (OR = 1.01, 95% CI = 1.00, 1.02).
An and Xiang 2015	U.S.	Cross-sectional	2,381,292	≥18	PM_2.5_	One unit (1 μg/m^3^) increase in county monthly average PM_2.5_ concentration was found to be associated with an increase in the odds of physical inactivity by 0.46% (OR = 1.0046, 95% CI = 1.0034, 1.0059).
Alahmari et al. (2015)	U.K.	Longitudinal	73	71.1 ± 8.7	PM_10_, O_3_	Relationship between PM_10_ (μg/m^3^) and daily step count: regression coefficient = -5.4 , 95% CI = -12.2, 1.3, p-value = 0.112. Relationship between O_3_ (μg/m^3^) and daily step count: regression coefficient = -8.0, 95% CI = -13.5, −2.4, p-value = 0.005. Relationship between PM_10_ (μg/m^3^) and hours spent outdoors: regression coefficient = 2.3 × 10^-3^, 95% CI = -6.5 × 10^-3^, 1.8 × 10^-3^, p-value = 0.275. Relationship between O_3_ (μg/m^3^) and hours spent outdoors: regression coefficient = -9.9 × 10^-3^, 95% CI = -14.2 × 10^-3^, −5.6 × 10^-3^, p-value < 0.001.
Chen and Lin 2016	China	Cross-sectional	2,268	42–49	General air quality	One-unit increase of perceived air quality is associated with a reduction in physical inactivity by 20% (OR = 0.80, 95% CI = 71%, 89%).
Yang and Zacharias 2016	China	Cross-sectional	852	≥60	General air quality	Air pollution, traffic safety, the lack of road space, climatic disadvantages, insufficient secure parking for bicycles, and inadequate night lighting are seen as major barriers by all commuters.
Hu et al. 2017	China	Cross-sectional	153	36.8 ± 7.9	General air quality	App users were less likely to participate in outdoor running, biking, and walking (F = 24.16, p < 0.01) when air pollution concentration increased.
Li and Kamargianni 2017	China	Cross-sectional	492	All ages	General air quality	In winter, biking (Coefficient = -0.009, t-Statistic = -2.63), bike-sharing (Coefficient = -0.058, t-Statistic = -6.71), and walking (Coefficient = -0.018, t-Statistic = -5.12) were not preferred when air pollution level increased. Instead travelers switched to the use of cars (Coefficient = 0.015, t-Statistic = 5.63), buses (Coefficient = 0.0002, t-Statistic = 0.06), taxis (Coefficient = 0.003, t-Statistic = 0.65), and electric bikes (Coefficient = 0.003, t-Statistic = 1.35). In summer, air pollution was negatively correlated with walking (Coefficient = -0.001, t-Statistic = -0.13) but positively correlated with biking (Coefficient = 0.016, t-Statistic = 1.85) and bike-sharing (Coefficient = 0.017, t-Statistic = 2.20).
Yu et al. 2017a	China	Longitudinal	848–890	66.8 (95% CI: 66.4–67.3)	PM_2.5_	An increase in ambient PM_2.5_ concentration by 1 standard deviation (56.6 µg/m^3^) was associated with a reduction in weekly total hours of walking by 4.69 (95% CI = 1.30, 8.08), a reduction in leisure-time Physical Activity Scale for the Elderly (PASE) score by 71.16 (95% CI = 28.91, 113.41), and a reduction in total PASE score by 110.67 (95% CI = 59.25, 162.08). An increase in ambient PM_2.5_ concentration by one standard deviation was associated with an increase in daily average hours of nighttime/daytime sleeping by 1.75 (95% CI = 1.24, 2.26).
Yu et al. 2017b	China	Longitudinal	3,223–3,242	18.2 ± 0.9	PM_2.5_	An increase in ambient PM_2.5_ concentration by one standard deviation (44.72 μg/m^3^) was associated with a reduction in 22.32 weekly minutes of vigorous physical activity (95% CI = 19.77, 24.88), a reduction in 10.63 weekly minutes of moderate physical activity (95% CI = 6.64, 14.61), a reduction in 32.45 (95% CI: 27.28, 37.63) weekly minutes of moderate to vigorous physical activity (MVPA), and a reduction in 226.14 (95% CI = 256.06, 196.21) weekly physical activity MET-minute scores.
An and Yu 2018	China	Longitudinal	12,184–12,291	18.1 (95% CI: 18.0–18.1)	PM_2.5_	An increase in the ambient PM_2.5_ concentration by one standard deviation (36.5 μg/m^3^) was associated with a reduction in weekly total minutes of walking by 7.3 (95% CI = 5.3, 9.4), a reduction in weekly total minutes of vigorous physical activity by 10.1 (95% CI = 8.5, 11.7), a reduction in daily average hours of sedentary behavior by 0.06 (95% CI = 0.02, 0.10).
Li and Kamargianni 2018	China	Cross-sectional	4,769	All ages	General air quality	Air pollution had significant negative effect on bike-sharing choice (Coefficient = -0.0045, t-Statistic = -8.29); Air pollution also had significant negative impact on walking (Coefficient = -0.0045, t-Statistic = -9.17), electric bike use (Coefficient = -0.0022, t-Statistic = -3.93), and bus use (Coefficient = -0.0020, t-Statistic = -2.65); Car-sharing (Coefficient = 0.0023, t-Statistic = 1.96) was the only transportation mode that had a positive correlation with air pollution level.
Zhang and An (2018)	China	Longitudinal	300		Air quality index	There was a negative non-linear relationship between air pollution level and television use. Compared to the days when air quality was good (0 ≤ AQI ≤ 50), days with fair air quality (50 < AQI ≤ 100), light air pollution (100 < AQI ≤ 150), and moderate-to-severe air pollution (AQI greater than 150) were associated with a reduction in daily average television use by 2.9 (p = 0.002), 4.6 (p < 0.001), and 1.9 (p = 0.369) minutes, respectively.
Zhao et al 2018	China	Cross-sectional	307	All ages	PM_2.5_	Residents with lower income (Coefficient = 0.58, 95% CI = -0.00, 1.16), those over 30 years old (Coefficient = 0.67, 95% CI = 0.11, 1.22), and male respondents were more likely to continue cycling in hazy weather.

AQI - air quality index; OR - Odds Ratio; CI - Confidence intervals.

### 3.1 Impact of air pollution levels on physical activity behaviour

An et al. (2018)’s systematic review synthesized the peer reviewed literature on impacts of air pollution on physical activity or sedentary behaviour for adults. Study designs included interventions or experiments, retrospective or prospective cohort studies, cross-sectional studies, and case-control studies. Of the seven studies (see Table 1) that met the inclusion criteria, six were conducted in the US, and one in the UK. All US-based studies adopted a cross-sectional study design, and the UK-based study adopted a prospective cohort design. Specific air pollutants assessed included particulate matter with aerodynamic diameter <2.5 and 10 µm (PM_2.5_ and PM_10_, respectively), O_3_, and oxides of nitrogen (NOx), whereas two studies focused on overall air quality. All studies found air pollution levels to be negatively associated with physical activity, with the meta-analysis of US studies revealing that one unit (μg/m^3^) increase in ambient PM_2.5_concentration was associated with an increase in the odds of physical inactivity by 1.1% among adults. Study participants, in particular those with respiratory disease, self-reported a reduction in outdoor activities to mitigate the detrimental impact of air pollution.

An et al. (2019) subsequently reviewed additional studies published on impacts of air pollution on health behaviours in China. Study designs included interventions or experiments, retrospective and prospective cohort studies, cross-sectional studies, and case-control studies. Search terms included studies from all LMICs, but only studies from China were identified. From the ten studies that met the inclusion criteria, six used a cross-sectional and four a prospective cohort design. Four studied a specific air pollutant and six focused on overall air quality using Air Quality Index (AQI). Decline in overall air quality and increase in PM_2.5_concentration was found to be associated with reduced daily/weekly duration of outdoor leisure-time and/or transportation-related physical activity such as walking, running and biking. In contrast, evidence linking overall air quality and PM_2.5_concentration to sedentary behaviour, such as watching TV, remained mixed and inconclusive. In conclusion, An et al. (2019) called for future studies that would use objective measures of physical activity and longitudinal/experimental design to examine the impact of air pollution on susceptible populations such as children and fragile older adults and residents in LMICs.

In UK, Smith et al. (2019) explored the associations between neighbourhood environmental characteristics and different physical activity outcomes in adults using cross-sectional data from the UK Biobank study. Higher concentration of NOx at home address was associated with lower levels of (n = 337 822) wrist-worn accelerometer (n = 65 967) measured physical activity, with the weakest association reported for total walking levels, when the model was adjusted for demographic and socioeconomic variables. For example, participants living in areas with the highest concentrations of air pollution had a lower levels of total physical activity.

### 3.2 Impact of air pollution episodes on physical activity behaviour

Daily changes in air pollution levels and media alerts informing the public about risky air pollution levels may alter day-to-day decisions on physical activity (Wen et al., 2009). In a cross-sectional telephone survey study from 33 888 adults in the United States, both media alerts and advices from medical professionals regarding air pollution were found to influence self-reported outdoor physical activity levels, with 16% and 31% prevalence decrease among adults without and with asthma (Wen et al., 2009). Saberian et al. (2017) estimated the impact of air quality alerts on daily cycling in Sydney, Australia, and observed a 14% to 35% decline in cycling levels among study participants following air pollution alerts. The effect tended to become weaker for the second consecutive day when the alert was issued, which authors hypothesised to be a consequence of an “alert fatigue”.

Choi et al. (2019) investigated the relationship between the daily concentration of PM_2.5_ and the number of visitors in recreational places in Seoul, South Korea, using mobile phone data to compare number of visitors in different categories of places (open spaces, commercial spaces, and indoor sport facilities). They concluded that when the level of PM increased, pedestrian volume decreased in open spaces, with number of visitors to mountains and hills, large parks, and waterfront parks decreased much more than the numbers of visitors to small parks. Commercial indoor spaces attracted more visitors with an increasing level of PM_2.5_, but the number of visitors to indoor sports facilities was unaffected by the level of PM_2.5_ (Choi et al., 2019). Similarly, in China, Jiang et al. (2019) found out that PM air pollution concentrations had negative impact on the maximum number of people visiting neighbourhood parks.

### 3.3 Remarks

The literature suggests that air pollution may discourage people from being physically active during high air-pollution days or may prevent people from engaging in physical activity overall in highly polluted environments. However, available evidence is based on relatively few studies, conducted mainly in the US and China, using mostly self-reported physical activity measures and among a healthy adult population. In addition, research is lacking on people’s perceptions of air pollution levels as well as on guidance related to physical activity behaviour during high air pollution levels. Especially, the impact of air pollution episodes for outdoor physical activity, independently or related to AQIs or alerts, remains uncertain. We found no evidence on impact of air pollution for indoor physical activity.

## 4 Exposure to air pollution while being engaged in physical activity

Physical activity in all domains (transport, leisure, occupation and domestic), both indoors and outdoors, takes place in microenvironments with some level of air pollution. Some of these environments could have higher air pollution concentrations than outdoor urban background environment (de Nazelle et al., 2017). Physical activity also changes the breathing pattern, leading to increases in to the inhalation of air pollution (Bigazzi and Figliozzi, 2014). Here we review the evidence on air pollution concentration in active travel microenvironments, and how changes in route of travel could reduce the exposure to air pollution. We also provide a brief overview on how inhalation and the dose of air pollution changes while being physically active, and what is known on air pollution concentration in other locations of physical activity.

### 4.1 Active travel and travel mode contrasts

A quantitative review of European studies measuring exposure while walking or cycling versus car or bus was published by de Nazelle et al. (2017). The review focused on studies that measured PM_2.5_, black carbon (BC), ultrafine particles (UFP) and carbon monoxide (CO) in the different travel modes with a simultaneous or quasi-simultaneous design (i.e. same time or nearly same time measurements in different modes). Pedestrians were consistently shown in this study to be the least exposed of the four modes. Ratios of bus, bike and car versus pedestrians were on average 1.3 to 1.5 for PM_2.5_, 1.1 to 1.7 times higher for UFP, and 1.3 to 2.9 times higher for CO. Cyclists were generally less exposed than car drivers on average, but equal for UFP. Differences between cyclists and bus users were inconsistent across studies. All modes had higher concentrations than background levels, in particular for PM_2.5_. These ratios are used in an updated version of the Health and Economic Assessment Tool for walking and cycling (HEAT), provided by the WHO Regional Office for Europe, to estimate mode-specific exposure to PM_2.5_ (Kahlmeier et al., 2017).

An unpublished update of these figures for a review of worldwide studies, however, paints a slightly different picture (Northover et al., 2017). Using the same methodology as de Nazelle et al. (2017), but with studies from around the world and a few more recent publications in Europe, Northover et al. (2017) preliminary results show that both cyclists and pedestrians are slightly more exposed than car occupants for PM_2.5_ (car to walk ratio 0.7, car to bike ratio 0.9). Other contrasts are not fundamentally different, although bus riders have more consistently higher exposure than cyclists for BC and CO. The car continues to be consistently more exposed than pedestrians for BC (two-fold) and CO (three-fold). Similarly, pedestrians and cyclists still have higher measured concentrations than background levels in the updated review (global average pedestrian to background ratios range from 1.2 for PM_2.5_to 4.0 for BC; cyclists versus background ranges from 1.7 for PM_2.5_to 3.2 for CO). The update additionally considered above ground and underground metro system concentrations, with the few available studies finding inconsistent results across pollutants.

### 4.2 Active travel exposure and route choice

Bigazzi and Figliozzi (2014) reviewed cyclist exposure in high versus low traffic routes and found that in high-traffic environments median exposure concentration was −4%, 39%, 26%, 47% 70% and 102% higher for PM_10_, BC, PM_2.5_, CO, UFP and volatile organic compounds (VOCs), respectively. The negative value for PM_10_ may seem surprising, however PM_10_ is a more homogenous pollutant than others listed, and the median is based only on 3 studies (compared to 5 to 11 for other pollutants). Studies published since Bigazzi and Figliozzi (2014) review are mostly in line with these findings (e.g. Good et al., 2015, MacNaughton et al., 2014). Further studies have modelled, instead of measured, exposure contrasts in different routes: Hertel et al. (2008) found 10 to 30% higher exposures in shortest versus less trafficked streets (NO_x_, NO_2_, CO, PM_10_, PM_2.5_); Hatzopoulou et al. (2013) estimated a 5% lower NO_2_ concentration on lowest exposure route vs shortest route; and Li et al. (2017) estimated a 50% lower PM_2.5_exposure in the cleanest vs shortest cycling path. Overall, studies are suggesting that it is better to take a longer but less polluted route than a shorter route with higher air pollution.

Few modelling studies have explored exposure contrasts for pedestrians. Mölter and Lindley (2015) found slightly lower exposures (around 1%) in the simulated lowest NO_2_ and PM_10_ exposure route versus the shortest routes to schools in Manchester, UK. Elford and Adams (2019) used a similar simulation model in Toronto, Canada, to estimate exposures to UFPs during school commutes, and concluded that for 86.6% of students, the shortest-route to school was the route that led to the lowest inhaled doses of (however this approach ignores the fact that commuters do not stop breathing when they arrive at their destinations. Li et al. (2017) found a 3.2% modelled reduction in inhaled dose for PM2.5 on the cleanest versus shortest pedestrian path, while for cycling dose was 50% smaller when using cleanest route.

### 4.3 Leisure-time physical activity

A limited number of studies have measured air pollution concentrations in places for leisure-time physical activity. Andrade et al. (2017)’s bibliographic review identified 34 studies measuring indoor air quality. Only two leisure-time physical activity locations have multiple studies from several locations: indoor ice arenas, with emissions from ice resurfacing machines (e.g. Pelham et al., 2002), and swimming pools, due to the high chlorine-containing air pollution in indoor air (e.g. Nemery et al., 2002).

Other measurement studies have concluded that particulate matter concentrations increase during the use in an indoor riding arena (Lühe et al., 2017), PM_10_ concentrations increase during classes in fitness centres (Ramos et al., 2014), and that during the use the PM_10_ concentrations increase more in indoor fronton, when compared to outdoor fronton (Alves et al., 2014).

### 4.4 Inhalation and dose of air pollution while engaged in physical activity

The dose of inhaled air pollution is critical to estimate the potential health risks of air pollution while doing physical activity, however this information is rarely available and typically not accounted for in large epidemiological studies. Pollutant inhalation rate, in mass per unit time, is the product of exposure concentration and inhalation rate (also called *ventilation rate* or *ventilation minute* or *pulmonary ventilation*), measured in volume of air per unit time (usually L/min). Various methods have been employed to assess inhalation rates, many of which were evaluated and compared in Dons et al. (2017). These include using fixed rates for an activity type or transforming available information into inhalation rates, such as activity type into a physical activity level (e.g. using Ainsworth et al., 2011 compendium of physical activity), and then into inhalation rates (e.g. stochastic equations developed by the EPA accounting for personal characteristics such as resting metabolic rate, age, gender and BMI (Johnson, 2002, de Nazelle et al., 2009)), or physiological measurements (heart rate) into inhalation rates (Dons et al., 2017). The different methods correlate relatively well and their choice depends on the type of information available (Dons et al., 2017).

Some authors have gone as far as modelling the actual deposition into the lung, and accounting for specific pollutant characteristics such as particle size and hygroscopicity, and gas solubility (Bigazzi and Figliozzi, 2014). Only a fraction of inhaled pollutants crosses the body boundary at the mouth and nose, and are either absorbed (gases) or deposited (particles) onto the respiratory tract or into the bloodstream. Absorbed/deposited pollutants are then either expelled or transported and incorporated into body tissues (Bigazzi and Figliozzi, 2014). Particle deposition and location of gas absorption in the respiratory tract are affected by breathing frequency, tidal volume, and fraction of oral breathing (Bigazzi and Figliozzi, 2014), which in-turn are influenced by the intensity of physical activity (ICRP, 2015). These changes in the breathing pattern could be quantified e.g. by applying the Human Respiratory Tract Model, which considers pollutant concentration, duration of exposure, breathing parameters, and chemical properties of the pollutant (ICRP, 2015).

Studies that have integrated inhaled dose (or deposition) when comparing travel modes have converged in their findings that, despite potentially lower air pollution concentration in active modes, once inhalation rates are taken into account, walking and cycling are at a clear disadvantage, when compared to other travel modes (Bigazzi and Figliozzi, 2014, de Nazelle et al., 2012, Nyhan et al., 2014). For example, in de Nazelle et al. (2012) inhaled 24 h dose (i.e. travel-related dose normalized to 24 h to account for different durations) was 1.4, 1.4 and 1.5 times higher for car occupant, pedestrian and cyclist, respectively, when compared to least exposure mode (bus). Many of the route choice studies reviewed above also pursue their analysis to compare cumulative exposures or inhalation dose of pollutants in different travel routes (Good et al., 2015, Li et al., 2017). Luo et al. (2018) in particular only report differences in intakes for different pedestrian routes (and not exposures), finding a 45 to 50% reduction in the lowest inhalation route compared to the shortest path for pedestrians, depending on time of day and willingness to deviate from shortest path (additional time travelling). Note also that few studies (Bigazzi, 2017, McNabola et al., 2007) have investigated trade-offs between walking/cycling speed and exposure concentration and duration, suggesting optimum commuting speeds (2–6 km/h for walking and 12–20 km/h for cycling; Bigazzi, 2017) to minimize air pollution inhalation.

The problem with the cumulative exposure and some of the inhaled dose studies is that they often fail to consider that people do not stop breathing once they arrive at their destination, hence hindering any fair comparison across modes or routes. Some, however, compared intake rates (per unit distance or unit time (Bigazzi and Figliozzi, 2014)), and one compared the lifestyle of pedestrian and cyclist commuters accounting for intake rates throughout the day (de Nazelle et al., 2012).

### 4.5 Remarks

Several studies have quantified PM_2.5_concentrations while cycling and walking for transport in high-income countries, especially in Europe, with the aim of predicting the public health effects of walking and cycling. However, a large amount of variability exists in the findings, and it remains uncertain whether these findings apply to LMICs and to other air pollutants, such as ozone and NO_2_. Studies measuring exposure contrast in transport microenvironments should further consider other modes such as metro systems, motorcycles, electric scooters and three-wheelers.

Furthermore, little is known for air pollution concentrations in locations where people regularly do physical activity for leisure (e.g. parks, indoor gyms). Current scientific literature does not provide enough quantitative evidence on air pollution concentration in places for leisure-time physical activity to assess the dose and exposure of air pollution while being physically active in those specific locations.

## 5 Epidemiology: Air pollution, physical activity and short-term health effects

Epidemiological evidence on air pollution and physical activity interaction in the real-world situation is divided between short-term studies, which measures the health outcomes (including acute surrogate or intermediate endpoints) hours or days after the exposure, and long-term studies, which follow the health outcomes for multiple years. A recent systematic review by Qin et al. (2019) provides a good overview of short-term literature, including experimental studies that tend to be before and after studies or taking place in chambers, and real-world case crossover studies that aim to disentangle the effects of air pollution and physical activity. In the below discussion we focus on the latter evidence. All mentioned studies are summarized in Table 2.

**Table 2:** Summary of short -term epidemiological studies that have combined health effects of air pollution and physical activity.

**Author**	**Year**	**Study Population**	**Location**	**Exposure length**	**Physical Activity (PA)**	**Air Pollutants**	**Outcome**	**Main findings**
Kubesch et al. (2015a)	2015a	28 healthy adults	Barcelona (Spain)	2-hours exposure in high and low TRAP^1^ environment	15 min intervals alternating rest and cycling on a stationary bicycle	BC UFP NO_x_ PM_10_ PM_2.5_ PM_coarse_	Systolic (SBP) and diastolic blood pressure (DBP)	Evidence of an interaction for PA and PM_10_ and PM_coarse_ increasing SBP
Kubesch et al. (2015b)	2015b	28 healthy adults	Barcelona (Spain)	2-hours exposure in high and low TRAP^1^ environment	15 min intervals alternating rest and cycling on a stationary bicycle	BC UFP NO_x_ PM_10_ PM_2.5_ PM_coarse_	Pulmonary function; systemic inflammation markers	No statistically significant evidence of an interaction.
Cole-Hunter et al. (2016)	2016	28 healthy adults	Barcelona (Spain)	2-hours exposure in high and low TRAP^1^ environment	15 min intervals alternating rest and cycling on a stationary bicycle	UFP BC PM_2.5_	Heart rate variability	PA reduced the negative impact of TRAP^1^ on heart rate variability in high TRAP site
Matt et al. (2016)	2016	29 healthy adults	Barcelona (Spain)	2-hours exposure in high and low TRAP^1^ environment	15 min intervals alternating rest and cycling on a stationary bicycle	BC UFP NOx NO PM10 PM2.5 PM_coarse_	Respiratory function	PA reduced the negative impact of TRAPP^1^ on respiratory function.
Sinharay et al. (2018)	2018	+ 60 yrs. with and without chronic lung or heart disease	London (United Kingdom)	2-hours	Walk	BC NO_2_ PM_10_ PM_2.5_ UFP	Cardiovascular and respiratory outcomes	Short term exposure to traffic pollution prevents the beneficial cardiopulmonary effects of walking for healthy individuals and individuals with ischemic heart diseases and COPD
Lovinsky-Desir et al. (2016)	2016	129 children aged 9–14 years	New York City (United States)	6 days	Moderate-to-vigorous physical activity	BC	Airway inflammation (FeNO)	Active children had less airway inflammation than non-active children, but primarily among children with lower personal BC exposure
Lovinsky-Desir et al. 2017	2017	135 children aged 9–14 years	New York City (United States)	6 days	Moderate-to-vigorous physical activity	BC	FOXP3 promoter methylation	Among children with high personal BC exposure, active children had lower FOXP3 methylation than non-active children. No evidence of association between MVPA and FOXP3 methylation among children with low personal BC concentration
Laeremans et al. (2018a)	2018a	122 healthy adults	Antwerp (Belgium), Barcelona (Spain), London (United Kingdom)	24 h	Physical activity energy expenditure (MET-hours)	BC	Heart rate variability Retinal vessel diameters Airway inflammation (FeNO) Lung function	No evidence of heart rate variability responses to physical activity, BC exposure or interaction was observed. FeNO and peak expiratory flow were detrimentally affected by BC regardless of PA levels
Laeremans et al.’s (2018b)	2018b	115 healthy adults	Antwerp (Belgium), Barcelona (Spain), London (United Kingdom)	7 days	Physical activity energy expenditure (MET-hours)	BC	Lung function	Physical activity was associated with improved pulmonary function at low BC concentrations, but benefits decreased when BC concentrations increased

TRAP - Traffic-related air pollution; FeNO - fractional exhaled nitric oxide.

### 5.1 Short-term exposures (up to 2 h)

To examine the interaction between short-term traffic-related air pollution (TRAP) and physical activity for cardiovascular outcomes, Kubesch et al. (2015a) conducted a crossover real-world exposure study comparing systolic (SBP) and diastolic blood pressure (DBP) responses to four different exposure scenarios: 2-h exposure in high or low-TRAP environment, both at rest and combined with intermittent moderate physical activity (15 min intervals alternating rest and cycling on a stationary bicycle). Exposure to high TRAP was associated with higher DBP (1.1 mm/Hg) post-exposure, irrespective of physical activity status. Physical activity lowered SBP more after exposure to the low-TRAP site (−2.3 mm/Hg) compared with the high-TRAP site (-1.6 mm/Hg). They only found evidence of an interaction between physical activity and both PM_10_ and PM coarse increasing SBP. Furthermore, Kubesch et al. (2015b) reported that intermittent physical activity compared to rest, irrespective of the TRAP exposure status, increased pulmonary function forced expiratory volume at one second (FEV1),(34 mL), forced vital capacity (FVC, the determination of the vital capacity from a maximally forced expiratory effort) (29 mL), forced expiratory flow (FEF25-75%) (91 mL)), lung inflammation (fraction of exhaled nitric oxide, FeNO, (0.89 ppb)), and systemic inflammation markers interleukin-6 (52.3%), leucocytes (9.7%) and neutrophils count (18.8%). Interquartile increases in coarse particulate matter were statistically significantly associated with increased FeNO (0.80 ppb) and neutrophil count (5.7%), while PM_2.5_ and PM_10_ increased leucocytes (5.1% and 4.0%, respectively). But again, they did not find a consistent evidence for an interaction between TRAP and physical activity for any of the outcomes of interest.

Cole-Hunter et al. (2016) reported that exposure to TRAP was associated with consistent decreases in heart rate variability (HRV); however, exposure–response relationships were not always linear over the broad range of exposures. For example, increase in black carbon was associated with a decrease in parasympathetic activity at the low-TRAP site, whereas no association was observed at the high-traffic site. Physical activity modified the impact of TRAP on HRV at the high-TRAP site, indicating that the physical activity may offset the impact of TRAP on parasympathetic modulation of the heart, particularly at higher exposure concentrations.

In a follow up study with a very similar design but different participants, Matt et al. (2016) found that physical activity (as 15-min intermittent cycle ergometry) was associated with a statistically significant increases of FEV1 (48.5 mL), FEV1/FVC) (0.64%) and (97.8 mL). An increase in exposure to one unit (1 μg/ m^3^) of PM coarse was associated with a decrease in FEV1(-1.31 mL) and FVC (-1.71 mL). For an otherwise equivalent exposure, an increase of physical activity by 1% of the maximum heart rate was found to reduce the immediate negative effects of particulate matter upon peak expiratory flow (PM_2.5_, 0.02 L/min; PM_10_, 0.02 L/min; PM coarse, 0.03 L/min) and the several hours delayed negative effects of particulate matter upon FVC (PM coarse, 0.11 mL). The negative impact of exposure to TRAP constituents on FEV1, FVC and peak expiratory flow was attenuated in those participants with higher TRAP pre-exposure levels. The authors concluded that associations between various pollutant exposures and respiratory measures are modified by the level of physical activity during exposure and TRAP pre-exposure of participants.

Sinharay et al. (2018) conducted a randomised, crossover study with people aged 60 years and older with stable ischaemic heart disease or chronic obstructive pulmonary disease (COPD), and age-matched healthy volunteers, who were randomly assigned to do a 2-hour walk either along a commercial street in London (Oxford Street) or in an urban park (Hyde Park). During the study, measured concentrations of black carbon, NO_2_, PM_10_, PM_2.5_, and ultrafine particles were significantly higher on Oxford Street than in Hyde Park. In all participants, irrespective of their disease status, walking in Hyde Park led to an increase in lung function (as measured by FEV1 and FVC) and a decrease in pulse wave velocity (PWV) and augmentation index up to 26 h after the walk. By contrast, these beneficial responses were attenuated after walking on Oxford Street. In participants with COPD, a reduction in FEV1 and FVC, and an increase in R5-20 (small airway resistance) were associated with an increase in during-walk concentration of NO_2_, ultrafine particles and PM_2.5_, and an increase in PWV and augmentation index with concentration of NO_2_ and ultrafine particles. In healthy volunteers, PWV and augmentation index were associated both with concentration of black carbon and ultrafine particles. The authors concluded that short-term exposure to traffic related air pollution prevents the short-term (0 to 24 h, depending on health outcome measured) beneficial cardiopulmonary effects of walking in people with COPD, ischaemic heart disease, and those free from chronic cardiopulmonary diseases.

### 5.2 Short-term exposures (days to weeks)

Lovinsky-Desir et al. (2016) performed a cross-sectional study nested in a birth cohort of African American and Dominican children living in the Bronx and Northern Manhattan, New York City. Children were recruited based on age presence (n = 70) or absence (n = 59) of current asthma. Children wore wrist mounted accelerometers for 6 days and were classified as 'active' if they had ≥ 60 min of moderate-to-vigorous physical activity (MVPA) each day and 'non-active' if they had < 60 min of MVPA on any given day, based on the US Center for Disease and Control and Prevention guidelines. Personal black carbon (BC) measured using a MicroAeth, was assessed during two 24-h periods, at the beginning and end of physical activity assessment, to estimate personal BC exposure. FeNO measurements were sampled at the beginning and end of the of physical activity assessment. After controlling for potential confounders, 'active' children had 25% higher personal BC exposure and 20% lower FeNO compared to 'non-active' children. Among children with high BC exposure, there was no relationship between activity and FeNO. The significant protective relationship between activity and airway inflammation was largely driven by children with lower BC exposure (n = 96). Authors concluded that children who live in an urban environment and who are physically active on a daily basis have higher personal exposure to BC, and that this exposure offsets the protective relationship between physical activity and airway inflammation. However, the association between physical activity and FeNO was not significant among children with asthma diagnosis.

In the same population, Lovinsky-Desir et al. 2017 examined the association between physical activity, BC and buccal cell DNA methylation of the forkhead box p3 (FOXP3) gene promoter, a proposed biomarker of Treg activity, which increases with exercise and suppresses airway inflammation, but decreases in association with exposure to air pollution. After controlling for confounders, authors found no significant relationship between physical activity and FOXP3 promoter methylation. However, in stratified analyses, among children with higher BC exposure (≥1200 ng/ m^3^), physical activity was associated with 2.37% lower methylation in promoter 2 but not among those with lower BC exposure (βestimate = 0.54%). Differences across strata were statistically significant (p for interaction = 0.04). After controlling for BC exposure, promoter 2 methylation was associated with reduced FEV1/FVC (βestimate = -0.40%) and reduced FEF25-75% (-1.46%). These findings suggest that physical activity may induce immunologic benefits, particularly for urban children with greater risk of impaired lung function due to exposure to higher air pollution.

Laeremans et al. (2018a) assessed the short-term (2-h and 24-h time window) effects of physical activity, air pollution and their interaction on a set of subclinical cardiovascular and respiratory outcomes in a panel of healthy adults. Participants from three European cities had their physical activity level and exposure to BC measured with wearable sensors during a week in three different seasons. At the end of each measurement week, health outcomes were evaluated. No evidence of heart rate variability responses to physical activity, BC exposure or interaction was observed during 24-h time window. FeNO and peak expiratory flow were detrimentally affected by BC regardless of PA levels

In the same population, Laeremans et al.’s (2018b) also assessed the impact of air pollution on the pulmonary health benefit of physical activity in the same population over a 3-week study period. For these analyses they averaged their exposure and health outcome measurements over 3 weeks on a participant level as a proxy for longer term exposure and lung function. They found negative interaction effects of physical activity and BC exposure on FEV1, FEV1/FVC and FEF25-75%. For BC concentrations up to approximately 1 μg/m^3^, an additional MET-hour per week resulted in a trend towards lung function increases (FEV1, FEV1/FVC and FEF25-75% increased 5.6 mL, 0.1% and 14.5 mL/s, respectively). This beneficial effect decreased in higher air pollution concentrations. Laeremans et al.’s (2018b) results suggest a greater need to reduce air pollution exposures during physical activity.

### 5.3 Remarks

Relatively few studies have assessed the combined short-term health effects of air pollution and physical activity in real world situations. Studies vary in design, exposure characteristics and outcomes studied, and it is not possible yet to draw some evidence-based conclusions. In general, there appears to be little evidence for interaction between air pollution and physical activity for short-term health effects, but there is suggestive evidence that short-term health effects of physical activity may be weaker or even non-existent at higher levels of air pollution.

## 6 Epidemiology: Air pollution, physical activity and long-term health effects

Long-term epidemiological studies follow the health outcomes years after the exposure to air pollution and physical activity were measured, with the aim to estimate long-term, or chronic, impact of cumulative exposure to different risk factors. In this review, we include studies that focus on the effect of physical activity on health outcomes, and its interaction with air pollution, or present joint effects of physical activity and air pollution on a health outcome, including interaction between two exposures (see Table 3).

**Table 3:** Summary of the long-term epidemiological studies that have combined health effects of air pollution and physical activity.

**Author**	**Year**	**Study Population**	**Location**	**Physical Activity (PA)**	**Air Pollutants**	**Outcome**	**Main findings**
McConell et al.	2002	3,535 children age 9–16 years	Southern California	Number of team sports played (0,1,2, ≥3)	PM_10_PM_2.5_ NO_2_ O_3_	Asthma incidence, parent reported	Increased risk of asthma when playing ≥ 3 sports (compared to 0 sports) in high O_3_ communities (OR:3.3; 95%CI: 1.9–5.8), and not in low O_3_ communities (OR:0.8; 95%CI: 0.4–1.6); no difference in PM
Yu et al.	2004	821 children age 8–12 years	Hong Kong	Habitual exercise (sports, free play, running, ball games, cycling)	SO_2_ NO_2_ PM_10_	Maximum oxygen intake (VO_2_ max)	Children who exercised in high pollution area had lower VO_2_ max than children who exercised in low pollution areas (27.9 vs. 29.8 mL.kg(-1).min(-1))
Andersen et al.	2015	57,000 adults, age 50–65 years	Denmark, Diet, Cancer and Health Cohort	Sport Cycling Walking Gardening	NO_2_	Mortality, CVD, respiratory, cancer, diabetes	Benefits of cycling, participating in sport, and gardening on all-cause and cause specific mortality similar in both, low and high NO_2_ areas
Fisher et al.	2016	57,000 adults, age 50–65 years,	Denmark, Diet, Cancer and Health Cohort	Sport Cycling Walking Gardening	NO_2_	Asthma, COPD	Benefits of cycling, participating in sport, and gardening on asthma and COPD incidence seen in both, low and high NO_2_ areas
Kubesch et al.	2018	57,000 adults, age 50–65 years,	Denmark, Diet, Cancer and Health Cohort	Sport Cycling Walking Gardening	NO_2_	Myocardial infarction (MI)	Benefits of cycling, participating in sport, and gardening on MI incidence seen in both, low and high NO_2_ areas
Fuertes et al.	2018	2,228 adults, age 27–67 years	Multi-centre ECRHS study, 14 countries	Leisure-time vigorous PA	PM_10_ PM_2.5_ NO_2_	Lung function (FEV_1_, FVC)	PA positively associated with FEV_1_/FVC in smokers, irrespective of air pollution; in never-smokers association negative in areas with high PM_10_
Zhang and An (2018)	2018	359,067 adults, age ≥ 18 years	Taiwan	Habitual PA (inactive, low, moderate, high)	PM_2.5_	Systemic inflammation, white blood cell (WBC)	Inverse association between PA and WBC; positive association between PM_2.5_ and WBC; no interaction between PA and PM_2.5_
Sun et al.	2019	66,820 adults, age ≥ 18 years	Hong Kong, Elderly Health Service Cohort	Habitual PA (walking, stretching, TCE, aerobic)	PM_2.5_	Mortality, CVD, respiratory	No interaction between PA and PM_2.5_; the beneficial effects of habitual PA outweighed the detrimental effects of long-term exposure to PM_2.5_ on mortality

ECRHS - European Community Respiratory Health Survey; TCE - traditional Chinese exercise; CVD - cardiovascular disease; FEV_1_ – forced expiratory volume in 1 s, FVC – forced vital capacity.

### 6.1 Children and adolescents

One of the earliest studies, from 2002, was based on a cohort of 3535 children, 9–16 years old, with no history of asthma, who were recruited in 1993 from schools in 12 communities in Southern California, and followed for 5 years for incidence of asthma (self-reported doctor diagnosis) (McConnell et al., 2002). Children’s parents reported physical activity as participation in sport teams in the last 12 months and number of sports played (0, 1, 2, ≥3). The communities were classified into low and high pollution, and 4-year measurements of O_3_, NO_2_, PM_2.5_ and PM_10_ were performed. Mean levels of O_3_, NO_2_, PM_2.5_ and PM_10_ in high vs. low pollution communities were: 38.5 vs. 25.1 ppb, 29.2 vs.10.8 µg/ m^3^, 21.4 vs. 7.6 µg/ m^3^, and 43.3 vs. 21.6 µg/ m^3^, respectively. McConnell et al. found that participating in team sports was associated with development of asthma in children residing in areas with high O_3_, and not in areas with low O_3_ levels, while the effect of participating in team sports on asthma did not differ by PM or NO_2_ levels (McConnell et al., 2002).

Using similar approach Yu et al. (2004) compared maximum oxygen intake (VO_2_max) between 821 children aged 8 to 12 from two districts of Hong Kong, defined as high and low air pollution districts. Parents of the children filled out the questionnaire on physical activities (frequency and duration of organized sports and vigorous free play, such as running, cycling, and ball games), whereas the VO_2_max, a proxy of cardiopulmonary fitness, was assessed once by a multistage fitness test (MFT). Air pollution was measured for a 12-month period for each district, during which mean SO_2_, NO_2_, and PM_10_ in high vs. low pollution district were: 22.8 vs. 11.8 µg/ m^3^, 58.5 vs. 42.9 µg/ m^3^, and 57.6 vs. 44.9 µg/ m^3^, respectively. The study concluded that children who exercised in high air pollution district had significantly lower VO_2_max than children who exercised in low air pollution areas (27.9 vs. 29.8 mL.kg(-1).min(-1), after adjusting e.g. for physical activity, disease symptoms and parents job) (Yu et al. 2004), suggesting that physical activity in a polluted environment might limit beneficial effect in cardiopulmonary fitness.

### 6.2 Adults

#### 6.2.1 Beneficial effects of physical activity modified by air pollution

Three studies based on the Danish Diet, Cancer and Health study, with over 50,000 participants recruited between 1993 and 1997, when they reported information on their physical activity (participation in sports, cycling, walking, gardening) used a prospective cohort design to estimate long-term effects (follow-up between 13 and 18 years) of leisure-time and utilitarian physical activities (cycling, participation in sport, walking, an gardening) on overall and cause specific mortality (Andersen et al., 2015), risk of asthma and COPD (Fisher et al., 2016), and risk of myocardial infarction (MI) (Kubesch et al., 2018). These studies are the first to apply individual estimates of air pollution, in terms of residential NO_2_, as a proxy of long-term exposure to air pollution, and examined whether NO_2_ modified beneficial effects of physical activity on mortality and cardio-respiratory morbidity.

In the first paper in the series, from 2015, Andersen et al. found that inverse associations of participation in sports, cycling, and gardening with total, cardiovascular, and diabetes mortality were not modified by NO_2_. However, they found that reductions in respiratory mortality associated with cycling and gardening were significantly higher in subjects who lived in areas with moderate/low NO_2_ [hazard ratio (HR) = 0.55; 95% confidence interval (CI): 0.42–0.72 and 0.55; 95% CI: 0.41–0.73, respectively] than in those who lived in areas with high NO_2_ levels (HR = 0.77; 95% CI: 0.54–1.11 and HR = 0.81; 95% CI: 0.55–1.18, p-interaction = 0.09 and 0.02, respectively). This finding suggested that for respiratory diseases, there might possibly be some reduction in beneficial effect of physical activity, when exercising in areas with high air pollution.

Fisher et al. (2016) further explored findings by Andersen et al. (2015) by considering incidence of chronic respiratory diseases, asthma and COPD. Authors defined incidence of asthma and COPD as first ever hospitalization for asthma or COPD, in asthma and COPD-free subjects, respectively. They also considered asthma and COP exacerbation, defined as a first hospital contact for asthma or COPD, in those who had asthma or COPD, respectively at study cohort baseline. Authors found inverse associations of participation in sports (HR: 0.85; 95% CI: 0.75–0.96) and cycling (0.85; 0.75–0.96) with incident asthma, and of participation in sports (0.82; 0.77–0.89), cycling (0.81; 0.76–0.87), gardening (0.88; 0.81–0.94) and walking (0.85; 0.75–0.95) with incident COPD admissions. They also found positive associations between NO_2_ and incident asthma (1.23; 1.04–1.47) and COPD (1.15; 1.03–1.27) hospitalizations (comparing ≥21.0 µg/ m^3^ to <14.3 µg/ m^3^). They found no interaction between associations of any physical activity and NO2 on incident asthma or COPD hospitalizations, or exacerbations, concluding that two exposures have independent effects.

Finally, Kubesch et al. (2018) used the same approach as the two above-mentioned studies to examine effect modification of an association between physical activity and MI by NO_2_ levels. Authors found inverse associations between participation in sports (HR; mean 0.85; 95% CI 0.79–0.92), cycling (0.91; 0.84–0.98), gardening (0.87; 0.80–0.95) and incident MI, while recurrent MI was not associated with cycling (0.80; 0.63–1.02), walking (0.82, 0.57–1.16), and gardening (0.91; 0.71–1.18). Authors found no effect modification of the associations between physical activity and MI by NO_2_ levels. These three papers document that in urban areas with relatively low air pollution levels, as Copenhagen (Denmark), benefits of physical activity seem to outweigh risk by additional exposure to air pollution during exercis but cannot be necessarily extrapolated to other urban areas with higher air pollution levels as well as higher physical activity levels.

In the multi-centre European Community Respiratory Health Survey study, Fuertes et al. (2018) examined whether a long-term beneficial effect of leisure-time physical activity on lung function in 25 to 67 years old subjects is modified by air pollution. They found that physical activity (defined as self-reported exercise of at least two times per week and for a duration of a at least 1 h a week) was positively associated with lung function in irrespective of air pollution levels at residence, showing that benefits of exercise outweighed harmful effects of air pollution (Fuertes et al., 2018).

#### 6.2.2 Joint effects of physical activity and air pollution

Zhang et al. (2018) studied joint effects of habitual physical activity (inactive, low, moderate and high) and long-term exposure to residential PM_2.5_ on white blood cell (WBC) count, marker of systemic inflammation, in 359,067 adults from a cohort of Taiwanese residents who participated in a standard medical examination from 2001 to 2014. Mean level of PM_2.5_ during study period was 26.5 µg/m^3^. Authors found a dose–response relationship between physical activity and WBC (decreasing WBC with increasing level of physical activity), in a model adjusted for PM_2.5_, as well as positive association between PM_2.5_ and WBC, at all levels of physical activity. They found no interaction between physical activity and PM_2.5_, concluding that effects of two exposures on systemic inflammation are independent of each other.

Sun et al. (2019) studied joint effects of air pollution and exercise on cardiovascular and respiratory mortality in elderly subjects (age 65 years and older) in Hong Kong, from the Elderly Heath Service Cohort. Authors found positive association between exercise and reduction in risk of cardiovascular and respiratory mortality and negative association between PM_2.5_ and cardiovascular mortality, as expected, but no interaction between physical activity and air pollution. Authors concluded that the beneficial mortality effects of habitual physical activity outweighed the detrimental effects of long-term exposure to air pollution in Hong Kong, where 50% of population was exposed to levels of PM_2.5_ higher than 35 µg/m^3^.

### 6.3 Remarks

The evidence on long-term health effects is mixed. Several studies suggest that there is no interaction between air pollution and physical activity on long-term health outcomes for mortality, incidence of asthma, COPD, and MI, and systemic inflammation markers (Andersen et al., 2015, Fisher et al., 2016, Kubesch et al., 2018, Zhang et al., 2018 Jan). Notably, all these studies are in low-exposure (Denmark) or medium-exposure (Taiwan) settings. A few studies suggest that physical activity may have a beneficial effect in protecting against adverse effects of air pollution on premature mortality and reduced lung function (Wong et al., 2007, Lamichhane et al., 2018). All these studies point in the direction that long-term benefits of physical activity in urban areas are not outweighed by risks related to excess exposure to air pollution during physical activity. However, two studies in children, both in relatively high air pollution settings (Southern California, mean O_3_ 75.4 ppb in high and 50.1 ppb in low level communities; Seoul study with mean PM_2.5_ of 33.4 µg/m^3^), suggest that exercising in areas with high air pollution may exacerbate risk of asthma (McConnell et al., 2002) and reduce exercise-related benefits in terms of cardio metabolic fitness (Yu et al. 2004), when compared to exercising in areas with lower air pollution. This may suggest that children may represent especially susceptible population to additional adverse effects of air pollution during physical activity and also that this relation may change depending on air pollution levels.

## 7 Public health modelling studies

A growing number of public health modelling studies have quantified risk–benefit trade-offs for transport-related physical activity and air pollution (Mueller et al., 2015, Doorley et al., July 2015). Most of the evidence is based on the comparative risk assessment method to quantify the change in the burden of disease attributable to changes in exposure to health risk factors.

Mueller et al. (2015) systematically reviewed health impact assessments of mode shifts to active travel and identified a total 30 studies that met the inclusion criteria. Except for two studies, all quantified the health effects of changes in physical activity and 17 studies quantified the health effects for changes in exposure to air pollution. The health benefits from physical activity outweighed the health harms from air pollution in all studies. All the studies, except one (Delhi, India, in Woodcock et al. 2009) included a study population outside the high-income countries. Since the review, de Sá et al. (2017) study from the Sao Paulo, Brazil, showed that in some active transport scenarios the negative health impact of air pollution was larger than the physical activity benefits, suggesting that the results from high-income countries might not be transferable to LMICs.

**Fig. 2:**
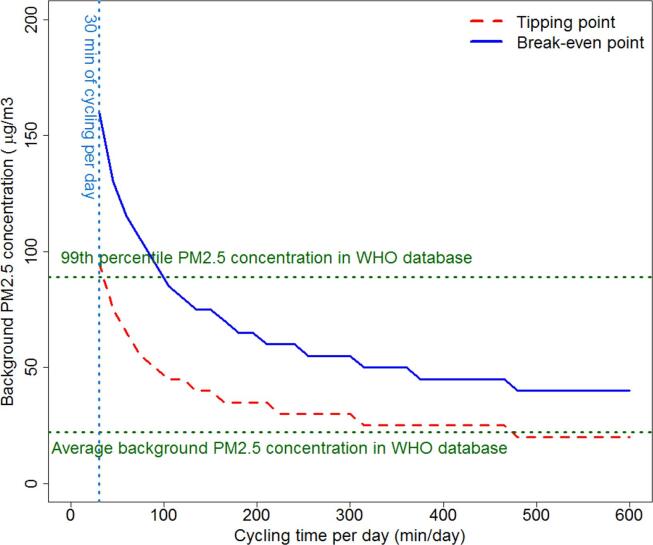
Tipping and break-even points for different levels of cycling (red dashed line and blue solid line, respectively) (minutes per day, x-axis) and for different background PM2.5 concentrations (y-axis). Green lines represent the average and 99th percentile background PM2.5 concentrations in World Health Organization (WHO) Ambient Air Pollution Database (World Health Organization (WHO), 2014). Figure . (For interpretation of the references to colour in this figure legend, the reader is referred to the web version of this article.)

Tainio et al. (2016) used a comparative risk assessment approach to examine the risk–benefit trade-off for cycling and walking in PM_2.5_ annual concentrations varying from 5 µg/ m^3^ to 200 µg/ m^3^, and separately in six most polluted cities in different world regions. The results were illustrated by calculating the “tipping point” and “break-even point”. At the “tipping point” an incremental increase in active travel would no longer lead to increased health benefits, while increasing active travel even more could lead to the “break-even point”, where the risk from air pollution started to outweigh the benefits of physical activity (see Fig. 2) . The study found that for the majority of the studied PM_2.5_ concentrations the benefits from physical activity (more walking or cycling) overweight the negative health impacts from air pollution exposure. For example, for the global average PM_2.5_ concentration in urban areas (22 µg/m^3^), based on cities included the World Health Organization (WHO) Ambient Air Pollution Database (WHO, 2014), the tipping point would be reached after 7 h of cycling every day of the year. However, in cities with PM_2.5_ concentrations above 100 µg/m^3^ the health risks of air pollution will overcome the benefits of physical activity much earlier, especially for regular cyclists (more than 1 h of cycling per day for PM_2.5_ concentration of 100 µg/m^3^).

adapted from Tainio et al. (2016)

Giallouros et al. (2020) extended Tainio et al. (2016) study by modelling risk–benefit trade-offs of avoiding high air pollution days, based on daily PM_2.5_ data from six cities (Helsinki, London, Sao Paulo, Warsaw, Beijing, New Delhi). In days with high air pollution, people were expected to travel by public transport or stay home. Health effects were estimates for annual average exposure to PM_2.5_ and physical activity. The study predicted that in all cities everyday walking and cycling was beneficial for health and that avoiding high air pollution days did not lead to health benefits. In two cities, Beijing and New Delhi, avoiding high air pollution days was expected to decrease benefits if threshold value for high air pollution was set for too low value (<100 µg/ m^3^).

### 7.1 Remarks

Public health modelling studies have indicated that the health benefits of physical activity usually outweigh the risks of air pollution in shifts towards walking, cycling and public transport in high income countries. Only few examples exist for LMICs, and beside Gupta and Elumalai (2017), none have quantified exposure to air pollution during leisure-time physical activity. Most of the modelling studies also tend to focus on PM_2.5_ air pollution rather than accounting for multiple pollutants (NO_2_, ozone).

## 8 Conclusions

Physical activity and air pollution are linked through multiple physiological and behavioural mechanisms, and these relations have important implications for public health, especially in locations with high air pollution concentrations, which are commonly locations also with higher levels of total physical activity. Some of the most relevant relations for public health have been discussed in specific chapters, and some initial recommendations for policy, public health and research are summarized in Table 4.

**Table 4:** Recommendations for policymakers, public health professionals and researcher.

**Policymakers and public health professionals**	**Researchers**
• Physical activity should be promoted and air pollution should be lowered; • Long-term benefits of physical activity seem to outweigh the risks of air pollution, supporting further development of policies that enable physical activity at local and global level; • Strategies to mitigate air pollution while being physically active should be considered when building e.g. sport facilities, parks, schools, kindergartens, and nurseries, especially when the facility is aimed for vulnerable people (e.g. children and adolescents, elderly, pregnant women or those with existing health conditions); • When developing active travel (walking, cycling) infrastructure, actions to mitigate the negative impact of air pollution caused by nearby motorized transport should be considered and acted upon to negate it e.g. by building through parks and green areas and/or away from busy roads; • Policies aimed towards reducing the harm of the air pollution episodes should consider both the short-term and long-term health consequences of air pollution and physical activity; • Communication about the benefits of physical activity and harms of air pollution should be tailored to different population subgroups; • Co-benefits and trade-offs should be considered through intersectoral collaboration in the development of policies and interventions to address air pollution and promote physical activity; and • Policies and interventions directly and indirectly linked to air pollution and physical activity (e.g. interventions on the built environment) should have their impact on both evaluated and monitored.	Additional research needed on: • the relation between air pollution and physical activity a) in LMICs; b) on intracity spatial variations, particularly in highly polluted sites, also for HIC; c) on vulnerable populations, such as children and adolescents, elderly, pregnant women, workers, and those with pre-existing health conditions; and d) for pollutants other than PM2.5 (e.g. UFP, BC, NO2, VOCs and ozone); • the impact of small daily increases in air pollution or living in polluted air on physical activity behaviour; • understanding daily, weekly and seasonal variation on overall exposure to air pollution when considering changes in ventilation rates caused by physical activity; • the risks and benefits of exercising in polluted air for non-transport related physical activity, for both outdoor and indoor environments; • effectiveness of school closure, sport event limitations and other similar interventions that aim to reduce harm of air pollution episodes; and • how to tailor communication and engagement on the relations between air pollution and physical activity for different target audiences. Finally, we need: • prospective studies on the relations between air pollution and physical activity using state-of-the-art individual measurements; • summarized evidence in the form of reviews and meta-analyses, particularly for short-term and long-term epidemiological studies, active travel HIAs and exposure contrast studies in transport microenvironments and other domains of physical activity practice; and • Systems thinking approaches and science around the implementation of measures to promote physical activity and address air pollution concomitantly.

HIA = Health Impact Assessment; LMIC = Low- and Middle-Income Countries; HIC: High-Income Countries; PM_2.5_ = Fine particulate matter; UFP: Ultrafine particles; BC = Black Carbon; NO_2_ = Nitrogen dioxin; VOC: volatile organic compounds.

Overall, epidemiological and modelling evidence suggests that the long-term benefits of physical activity in urban areas outweighed the risks from exposure to air pollution. Pedestrians were consistently shown to be the least exposed to air pollution when compared to all other modes of transport. Air pollution and physical activity seem to work independently on metabolic pathways to health. There is some suggestive evidence that short-term health effects of physical activity may be weaker or even non-existent at higher levels of air pollution in vulnerable populations – those with cardiovascular or respiratory diseases. Moreover, air pollution levels and alert systems seem to discourage physical activity behaviour.

This review also highlighted some significant open questions in assessing and better understanding the global health impact of the relations between physical activity and air pollution. For instance, the results show that there is scarce evidence in LMICs across all potential relations between physical activity and air pollution – most of which is presently coming from China. This is particularly relevant in the context of LMICs which tend to have air pollution levels many times over the WHO guideline value as well as higher levels of physical activity – mainly not by choice but rather as a living necessity. Moreover, some LMICs currently face a rapid increase in urbanisation and motorisation, a context wherein better evidence on the links between air pollution and physical activity is much needed. There is also very limited evidence on the air pollution and physical activity relations on potentially more sensitive population subgroups, such as children, elderly, pregnant women and people with pre-existing conditions.

Most of the reviewed studies used self-reported measures of physical activity with widely varying methods combined with retrospective data on air pollution levels. Studies prospectively and simultaneously assessing physical activity and air pollution levels as well as increased exposure to air pollution while physically active might offer a better overview on how these two are related. More information is also needed on air pollution concentrations from indoor physical activity locations to better assess the potential impact of air pollution in settings dedicated to physical activity.

Future research on interventions related to air pollution and physical activity – such as on built environment – should consider the whole range of effects that are likely to affect the population, in order to maximise short- and long-term health gains while also ensuring that possible negative effects are anticipated and minimised. For instance, existing research supports a positive association between built environment attributes (density, land-use mix, street connectivity, access to public transport, park density) and overall physical activity (e.g. McCormack and Shiell, 2011, Sallis et al., 2016), while several of the same attributes are also associated with air pollution concentration and exposure (Goenka and Andersen, 2016 Dec 10, Hunter et al., 2015 Jan, Giles-Corti et al., 2016 Dec 10, Stevenson et al., 2016 Dec 10, Markevych et al., 2017, Nieuwenhuijsen, 2018 Jul).

At the same time, interventions on the built environment are context-specific and so will likely be the potential impact of these interventions on air pollution and physical activity, which is particularly relevant for evidence on potential solutions related to city compactness, transport systems and green space, to name a few (Goenka and Andersen, 2016 Dec 10, Hunter et al., 2015 Jan, Giles-Corti et al., 2016 Dec 10, Stevenson et al., 2016 Dec 10, Markevych et al., 2017, Nieuwenhuijsen, 2018 Jul).

The strength of this review is the wide scope with focus on many relevant but less studied topics in the air pollution and physical activity links as well as its global coverage, and the identification of research gaps (Table 4). We acknowledge the lack of systematic review methods in the exploration of the literature for each research area identified; however, this did not preclude us of providing a comprehensive landscape of the state-of-art and research gaps of the field, which were the main objectives of this work. This review did not consider non-academic grey literature nor occupational studies, which could have provided additional insights for all the research areas addressed.

Overall, this review calls for international collaboration between air pollution and physical activity research fields to better understand the multiple relations between air pollution and physical activity, from the assessment of the physical activity benefits lost due to air pollution to how different policies and interventions impacting both physical activity and air pollution levels can reduce risks and deliver health gains.

## Declaration of Competing Interest

The authors declare that they have no known competing financial interests or personal relationships that could have appeared to influence the work reported in this paper.

## Figures and Tables

**Table:** 

AP	Air pollution
AQI	Air Quality Index
BC	black carbon
CI	confidence interval
CO	carbon monoxide
COPD	chronic obstructive pulmonary disease
CVD	cardiovascular disease
DBP	diastolic blood pressure
ECRHS	European Community Respiratory Health Survey
FEF	forced expiratory flow
FeNO	fraction of exhaled nitric oxide
FEV1	forced expiratory volume at one second
FOXP3	forkhead box p3
FVC	forced vital capacity
HEAT	Health and Economic Assessment Tool for walking and cycling
HIA	Health Impact Assessment
HIC	High Income Countries
HRV	heart rate variability
IHD	ischemic heart disease
ISEE	International Society for Environmental Epidemiology
ISES	International Society of Exposure Science
ISPAH	International Society for Physical Activity and Health
LMICs	low- and middle-income countries
MFT	multistage fitness test
MI	myocardial infarction
MVPA	moderate-to-vigorous physical activity
NCDs	non-communicable diseases
NO2	nitrogen dioxide
NOx	oxides of nitrogen
O3	ozone
PA	physical activity
PASE	Physical Activity Scale for the Elderly
PM	particulate matter
PWV	pulse wave velocity
SBP	systolic blood pressure
TCE	traditional Chinese exercise
TRAP	traffic-related air pollution
UFP	ultrafine particles
VO2	maximum oxygen intake
VOCs	volatile organic compounds
WBC	white blood cell
WHO	World Health Organization
